# Remdesivir Alleviates Acute Kidney Injury by Inhibiting the Activation of NLRP3 Inflammasome

**DOI:** 10.3389/fimmu.2021.652446

**Published:** 2021-05-21

**Authors:** Liang Yin, Haoxin Zhao, Huiyu Zhang, Yi Li, Yuhao Dong, Huijin Ju, Feng Kong, Shengtian Zhao

**Affiliations:** ^1^ Department of Central Laboratory, Shandong Provincial Hospital Affiliated to Shandong First Medical University, Jinan, China; ^2^ Engineering Laboratory of Urinary Organ and Functional Reconstruction of Shandong Province, Shandong Provincial Hospital affiliated to Shandong First Medical University, Jinan, China; ^3^ Department of Pathology, Case Western Reserve University School of Medicine, Cleveland, OH, United States; ^4^ Department of Urology, Shandong Provincial Hospital Affiliated to Shandong University, Jinan, China; ^5^ Department of Urology, Shandong Provincial Hospital Affiliated to Shandong First Medical University, Jinan, China; ^6^ Binzhou Medical University, Yantai, China

**Keywords:** remdesivir, NLRP3 inflammasome or NOD-, LRR-, pyrin domain-containing protein 3 (NLRP3)inflammasome, NF-κB, macrophage, acute kidney injury

## Abstract

Acute kidney injury (AKI) is a frequent clinical complication in critically ill patients, and it rapidly develops into renal failure with high morbidity and mortality. However, other than dialysis, no effective therapeutic interventions can offer reliable treatment to limit renal injury and improve survival. Here, we firstly reported that remdesivir (RDV, GS-5734), a broad-spectrum antiviral nucleotide prodrug, alleviated AKI by specifically inhibiting NOD-, LRR-, and pyrin domain-containing protein 3 (NLRP3) inflammasome activation in macrophages. Mechanically, RDV effectively suppressed the activities of nuclear transcription factor (NF)-κB, mitogen-activated protein kinase (MAPK), which further led to the reduction of the inflammasome genes of NLRP3 transcription, limiting the activation of NLRP3 inflammasome *in vivo* and *in vitro*. RDV also inhibited other pro-inflammatory genes including tumor necrosis factor-α (TNF-α), interleukin-6 (IL-6), IL-12, IL-1β, and interferon–β (IFN-β), leading to the reduction of inflammatory factors release. Thus, RDV can ameliorate AKI *via* modulating macrophage inflammasome activation and inflammatory immune responses and may have a therapeutic potential for patients with AKI in clinical application.

## Introduction

Acute kidney injury (AKI), one of the most common critical illnesses, is mainly characterized by a rapid decline in renal function, including a sudden decrease in glomerular filtration and an increase in serum creatinine concentration (SCr) or oliguria. This condition remains a significant clinical concern because of the high mortality, morbidity and treatment cost. AKI occurs frequently in septic patients with strong inflammation storm which increases mortality by six to eight fold. However, the mechanism underlying sepsis-caused kidney injury remains unknown. Thus, specific and effective therapeutic methods in clinical setting are currently lacking (1, 2).

Recent evidence shows that inflammation-induced organ damage is a fundamental pathophysiological mechanism explaining the development of sepsis-induced AKI. The infiltration of macrophages act as a key player in renal injury, inflammation and fibrosis, which contribute to disease progress (3). Initially, monocytes are recruited into the pathological kidney and differentiate into macrophages, which will be polarized to different phenotypes in response to the local microenvironment. Macrophages accumulate in the diseased kidney, differentiate into pro-inflammatory types majorly contributing to inflammation, fibrosis, and tubular injury and resulting in further kidney damage (4, 5). In human studies, the degree of macrophage infiltration positively correlates with the severity of kidney injury in patients with glomerulonephritis, suggesting the pathogenic role of macrophages in kidney disease. Inflammatory response is dependent on the activation of Toll-like receptor-derived signaling cascades (e.g., nuclear factor (NF)-κB, mitogen-activated protein kinase (MAPK), and interferon (IFN) regulatory factor 3) in macrophages, which initiate inflammatory cytokine expression (e.g., TNF-α, IL-6, IL-12, and IL-1β) and affect kidney inflammatory balance. Of note, NOD-, LRR-, and pyrin domain-containing protein 3 (NLRP3) inflammasome, as a critical innate immunity component plays critical roles in orchestrating host immunity homeostasis. The NLRP3 inflammasome consists of an intracellular sensor NLRP3, an adapter protein (apoptosis-associated speck-like protein, ASC) and an effector caspase-1 (6, 7). The assembly of NLRP3 inflammasome is triggered by receipt of a priming signal (signal 1) provided by the activation of cytokines or pathogen-associated molecular patterns (PAMPs), such as LPS, leading to NLRP3 transcription. Further, an activation signal (signal 2) provided by numerous PAMPs and damage-associated molecular patterns (DAMPs) including ATP and crystals activate multiple cellular signaling events including K^+^ efflux, Ca^2+^ flux, lysosomal disruption, mitochondrial reactive oxygen species (mtROS) production and mitochondrial DNA (mtDNA) release. The inflammasome activated caspase 1 cleaves pro-IL-1β and pro-IL-18, triggering inflammation (7, 8). Multiple inflammatory diseases including sepsis-induced AKI are involved in aberrant NLRP3 inflammasome activation. Thus, NLRP3 inhibition is an important emerging strategy for the treatment of the above disease. Nevertheless, the inhibitory mechanism and precise inhibitors targeting NLRP3 are not fully elucidated, presenting a major challenge (9). Hence, novel and specific NLRP3 inhibitors need to be identified to solve the potential risks such as off-target and side effects.

Remdesivir (RDV, GS-5734), as a broad-spectrum antiviral nucleotide prodrug with a promising *in vitro* antiviral activity, inhibits viral loads and improves disease outcomes in severe acute respiratory syndrome-associated coronavirus infected mice with critical inflammatory response (9, 10). Recently, the Food and Drug Administration has approved the clinical application of RDV in the treatment of COVID-19 patients, attracting many groups on the exploring the effect of RDV on other diseases. RDV exhibited protective effects against acute lung injury in rodent animals by reducing neutrophil infiltration and IFN level (11–13). RDV can suppress the expression of STING and attenuates high fat diet induced nonalcoholic fatty liver disease by regulating hepatocyte dyslipidemia and inflammation, which suggest the effect of RDV on inflammation-resolution in these diseases (10). However, the underlying mechanism remains unknown and the roles of RDV in other inflammatory diseases remain unexplored. Here we explored the possible roles of RDV in sepsis-induced AKI, especially focusing on its roles in NLRP3 inflammasome activation, to identify a novel approach for inflammatory disease treatment.

In the current study, we presented that RDV could effectively inhibit inflammatory immune responses, which specifically repress NLRP3 inflammasome activation in lipopolysaccharide (LPS)-activated macrophages in particularly by inhibiting NF-κB and MAPK pathway. We propose that RDV has potential to serve as a therapeutic option for controlling the development of AKI.

## Materials and Methods

### Materials

RDV-(GS-5734) (purity > 99.5%) was purchased from *Selleck*, China. LPS was obtained from Escherichia coli O55:B5 (Sigma-Aldrich, China). Blood urea nitrogen (BUN), SCr assay kit and mouse albumin enzyme-linked immunosorbent assay (ELISA) kit were supplied by Wuhan Saiweier Bioengineering Institute, China. Mouse ELISA determination kits of TNF-α, IL-6, IL-1β, IL-12 were purchased from *Invitrogen*. Rabbit monoclonal antibodies to ERK (1:1000 dilution, #4695), p38 (1:1000 dilution, #8690), p-ERK (1:1000 dilution, #4370), p-p38 (1:1000 dilution, #4511), IκBα (1:1000 dilution,#4814), p-IκBα (1:1000 dilution, #2859), NF-κB p65 (1:1000 dilution, #8242), p-p65 (1:1000 dilution, #3033), AIM2 (1:1000 dilution, #63660S), Cleaved-IL-1β (1:1000 dilution, #63124), ASC (1:1000 dilution, #67824), Caspase-3 (1:1000 dilution, #14220), Cleaved Caspase-3 (1:1000, #9664), and murine monoclonal antibody to pro-IL-1β (1:1000 dilution, #12242), were purchased from *Cell Signaling Technology (CST)*, USA. Mouse monoclonal antibody to NLRP3 (1:2000 dilution, AG-20B-0014), Caspase-1 (1:2000 dilution, AG-20B-0042-C100), were obtained from *Adipogen*, USA. Rabbit monoclonal antibody to NLR family CARD domain-containing protein 4 (NLRC4) (1:1000 dilution, ab201792), GSDMD (1:1000 dilution, ab209845), goat Anti-Mouse IgG H&L (Alexa Fluor^®^ 647) (1:200 dilution, ab150115), and goat Anti-Mouse IgG H&L (Alexa Fluor^®^488) (1:200 dilution, ab150113) were acquired form *Abcam*, UK. DAPI (1:100 dilution, C0065) was obtained from *Solarbio*, China. HRP conjugated goat anti-rabbit IgG, HRP conjugated goat anti-mouse IgG and Mouse GAPDH (1:2000 dilution, 60004-1-Ig) were provided by *Beijing Zhongshan Golden Bridge Biological Technology*, China.

### Cell Culture and Treatment

To obtain mouse primary peritoneal macrophages, C57BL/6J mice (4–6 weeks old) were injected intraperitoneally with 3% Brewer’s thioglycollate broth (7, 8). Three days later, peritoneal exudate cells were harvested and incubated. Two hours later, nonadherent cells were discarded and adherent monolayer cells were used as peritoneal macrophages. Murine macrophage cell line RAW264.7 and RAW264.7-RFP-ASC stable cell line were cultured in Dulbecco’s Modified Eagle’s medium (DMEM) supplemented with 10% fetal calf serum, 10 mM glutamine, 100 U/ml penicillin, and 0.1 mg/ml streptomycin (Invitrogen, Carlsbad, CA, USA) in a humidified incubator at 37°C with 5% CO_2_ (14).

### Cell Stimulation

To analyze the effect of RDV on macrophages, cells were sorted into LPS and LPS+RDV group. Cells in the LPS group were cultured in the culture medium with LPS (100 ng/ml) and DMSO (same value with RDV) stimulation at different time points. Cells in the LPS plus RDV group were treated with LPS (100 ng/ml) and RDV (10 µM) at different time points. LPS and RDV were added into cells at the same time (10).

### Animals and Experimental Design

C57BL/6 male mice weighing 18 to 20 g were purchased from Jinan Pengyue Experimental Animal Breeding Co., Ltd. (Shandong). The animal care and experimental procedures were conducted in accordance with the Guide for the Care and Use of Laboratory Animals, and the protocol of this study was approved by the Institutional Animal Care and Use Committee of Shandong Provincial Hospital. All animal experiments were performed in accordance with the laboratory animal ARRIVE guidelines of the Laboratory Animal Ethical Commission of Shandong provincial Hospital. The *in vivo* experiments adhere to the guidelines for Minimal Quality Thereshold for Preclinical Sepsis Studies (MQTiPPS) (15, 16). A total of 1 ml working solution with a concentration 5 mg/ml was prepared: In 50 µl dimethyl sulfoxide stock solution, 400 µl PEG300 was added. The mixture was mixed well until it clarified. Then, 50 µl Tween 80 was added, and the mixture was mixed well until the solution became clear. Finally, 500 µl double-distilled water was added, and the solution was mixed well. Mice in the control and LPS groups were administered with an equal volume of normal saline by subcutaneous injection. Two ways of RDV administration we adopted: RDV pretreatment and RDV intervention one hour after challenge with LPS. During RDV pretreatment, mice were randomly divided into four groups (n = 6 in each group): control, RDV, LPS, and RDV + LPS groups. The method of RDV administration was according to the pre-experiment and previous reports. Mice in RDV and RDV +LPS groups were administered with RDV at a dosage of 25 mg/kg every 12 h for 7 days by subcutaneous injection (13, 17). After 7 days of continuous subcutaneous injection, mice in the LPS and RDV + LPS groups were i.p. injected with 10 mg/kg LPS to induce AKI. Mice in the control and RDV groups were injected with an equal volume of normal saline to the peritoneal cavity. After LPS injection for 12 h, the mice were sacrificed and kidney tissue and blood were collected for evaluation of kidney inflammation score and detection of serum cytokines (IL-1β, TNF-α, IL-12, and IL-6) by ELISA (18–20). During RDV intervention one hour post LPS challenge, mice were also randomly divided into four groups (n = 6 in each group): control, RDV, LPS, and RDV + LPS groups. Mice in RDV and RDV +LPS groups were administered with RDV at a dosage of 25 mg/kg post 10 mg/kg LPS challenge one hour by subcutaneous injection (21). After LPS injection for 12 h, the mice were sacrificed and kidney tissue was collected for evaluation of kidney inflammation score.

### Pathological Changes in Kidney Tissues

Kidney tissues were fixed with 4% paraformaldehyde for more than 2 days and then subjected to alcohol gradient dehydration and paraffin immersion and embedding. Paraffin-embedded renal tissues were cut into 5-μm sections for hematoxylin and eosin (HE) staining. Pathological changes in the kidney tissues, such as destruction or dilation of tubular structures and loss of the brush boarder, were observed under an optical microscope (TissueFAXS plus, ZEISS) (20).

### Detection of SCr, BUN, and Albumin Levels

Renal function status was evaluated by detecting changes in SCr and BUN and urinary albumin levels (18–20).

*Blood samples.* The blood samples were collected from the lower eyelid and eyeball allowed to stand for 30 min and then centrifuged at 4000 rpm for 20 min at 4°C. The supernatant was collected and used to determine SCr and BUN levels in accordance with to the instructions of the corresponding ELISA kits. SCr and BUN contents were expressed as milligrams per liter (mg/L).

*Urine samples.* Mice were placed in individual metabolic cages and provided with drinking water only. The urine collected from the mice was centrifuged within 24 h and centrifuged at 800 g for 10 min at 4°C. The supernatant was collected and used to determine albumin contents following the instructions of the mouse urinary albumin kit. Urinary albumin content was measured as nanograms per milliliter (ng/ml).

### Western Blot Analysis

After LPS stimulation for 12 h, the mice were sacrificed and kidneys were obtained and stored in a refrigerator at −80°C. Protein in kidneys was extracted following the instructions of a protein extraction kit (Beyotime, China), and protein concentrations were measured with a BCA protein assay kit (Sigma). The protein was separated by SDS–PAGE and transferred onto polyvinylidene fluoride (PVDF) membranes. The membranes were incubated with 5% milk for 2 h at room temperature, added with primary antibodies (nonphosphorylated forms of NF-κB p65, ERK, p38, IκBα; phosphorylated forms of ERK, p65, p38, p-IκBα), and placed in a refrigerator overnight at 4°C. The PVDF membranes were then washed thrice with Tris-buffered saline with 0.1% Tween ^®^ 20 (TBST) and incubated with secondary antibodies for 2 h at room temperature. Finally, the PVDF membranes were also washed with TBST and were added with developer solution and photographed. Protein ratios were normalized against GAPDH, and protein bands intensities were quantitatively analyzed by using Image J.

### Isolation of RNA and Quantitative Real-Time PCR

Total RNA was extracted with TRIzol reagent (Invitrogen) following the manufacturer’s instructions. Total RNA was reverse transcribed with oligo-dT primers into cDNA and then subjected to SYBR green RT-PCR to detect the expression of different molecules. Real-time primers were designed, and the following sequences were used: Murine NLRP3 sense: TGGATGGGTTTGCTGGGAT, antisense: CTGCGTGTAGCGACTGTTGAG. Murine TNF-α sense: GCCACCACGCTCTTCTGTCT, antisense: TGAGGGTCTGGGCCATAGAAC. Murine IL-6 sense: ACAACCACGGCCTTCCCTAC, antisense: CATTTCCACGATTTCCCAGA. Murine IL-1β sense: ACCTTCCAGGATGAGGACATGA, antisense: AACGTCACACACCAGCAGGTTA. Murine GAPDH sense: CGTCCCGTAGACAAAATGGT, antisense: TTGATGGCAACAATCTCCAC. Murine IL-12 sense: GGAAGCACGGCAGCAGAATA, antisense: AACTTGAGGGAGAAGTAGGAATGG. Murine iNOS sense: GAGCTCGGGTTGAAGTGGTATG. antisense: GAAACTATGGAGCACAGCCACAT. Gene expressions were analyzed using the 2^−ΔΔCT^ method.

### Immunofluorescence Staining and Confocal Analysis

Mouse primary peritoneal macrophages were primed with LPS (Sigma-Aldrich, 100 ng/ml) and LPS + RDV (Selleck, 10 µM), followed by ATP (Sigma-Aldrich, 5 mM) for 40 min. RFP-ASC speck formation was detected by fluorescence microscopy to evaluate the NLRP3 inflammasome activation and ASC speck formation assay (14). Mouse primary peritoneal macrophages were stimulated with LPS and LPS + RDV for 12 h. The cells were stained with a secondary antibody conjugated to Alexa Fluor 647 and nuclei were stained with DAPI (Molecular Probes, Invitrogen). The cells were examined by confocal laser microscopy (TCS SP8, Leica) (8). To detect NLRP3 levels in the kidney of AKI animal model, 5-μm tissue sections were deparaffinized, hydrated, subjected to antigen retrieval. The sections were washed with PBS and incubated with goat serum, and then treated with primary antibodies (NLRP3) overnight at 4°C. Thereafter, the sections were washed with PBS and incubated with FITC-conjugated secondary antibodies for 30 min at room temperature. Finally, the cell nuclei was stained with DAPI (20). Images were obtained by using a fluorescence microscope (TissueFAXS plus, ZEISS).

### Immunohistochemistry

Immunohistochemistry was performed as previously described on paraffin-embedded 5-µm tissue sections (22). Rabbit monoclonal F4/80 antibody (1:100 dilution, #30325) was purchased from Cell Signaling Technology (CST), USA.

### Statistical Analysis

All data was expressed as mean ± standard deviation. Two groups were compared by a paired Student’s t test, whereas multiple groups (> 2 groups) were compared with one-way ANOVA followed by Tukey’s *post hoc* test. Difference at P < 0.05 was considered statistically significant. All statistical analyses employed were performed using Prism 8.0.

## Results

### Effects of RDV on LPS-Induced NLRP3 Inflammasome Activation

**Figure 1:**
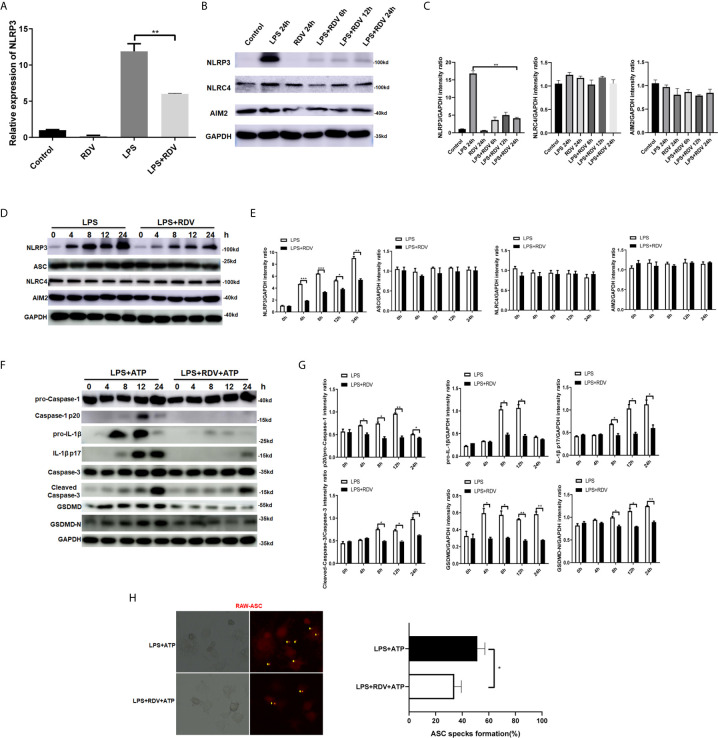
RDV inhibits the activation of NLRP3 inflammasome. **(A)** RT-PCR analysis of NLRP3 mRNA expression in mouse peritoneal macrophages stimulated with LPS, RDV, and LPS + RDV for 12 h. **(B)** Western blot analysis of NLRP3, NLRC4 and AIM2 in mouse peritoneal macrophages under LPS, RDV and LPS+RDV stimulation. **(C)** Relative intensity analysis of NLRP3, NLRC4 and AIM2. **(D)** Western blot analysis of NLRP3, ASC, NLRC4, AIM2 in mouse peritoneal macrophages under LPS and LPS+RDV stimulation at different time points. **(E)** Relative intensity analysis of NLRP3, ASC, NLRC4 and AIM2. **(F)** Western blot analysis of pro-Capase-1, Caspase-1, pro-IL-1β, IL-1β, Caspase-3, Cleaved-Capase-3, GSDMD, GSDMD-N. **(G)** Relative intensity analysis of Caspase-1/pro-Capase-1, pro-IL-1β/GAPDH, IL-1β/GAPDH, Cleaved-Capase-3/Caspase-3, GSDMD/GAPDH, GSDMD-N/GAPDH. **(H)** ASC specks formation assay of the effect of LPS and LPS + RDV stimulation for 12 h and treatment with ATP in the last 40 min. Percentages of ASC speck formation marked on the images. Data are representative as mean values ± SD from three independent experiments. *P < 0.05; **P < 0.01; ***P < 0.001.

Given that cellular NLRP3 level is vital for the assembly and activation of NLRP3 inflammasome, we first examined the NLRP3 expression by RDV treatment in LPS-activated macrophages. The mRNA level of NLRP3 in LPS +RDV was markedly lower than that in the LPS alone group (**Figure 1A**). Consistently, the protein level of NLRP3 was considerably decreased by RDV supplement (**Figures 1B–E** **) (** **Supplementary Figure 1**). Notably, RDV had no effect on the expression of absent in melanoma 2 (AIM2) and NLRC4, and two other pattern recognition receptors that are also involved in inflammasome formation (**Figures 1B–E**). In addition, RDV did not influence ASC level (**Figures 1D, E**), indicating that RDV selectively inhibited NLRP3 expression at the mRNA and protein levels. RDV also suppressed the expression of other inflammasome related genes, such as cleaved-Caspase-1, pro-IL-1β, cleaved-IL-1β, GSDMD, GSDMD-N, but RDV has no effect on pro-caspase-1 (**Figures 1F, G**). As a hallmark of inflammasome activation, we next determined whether the ASC speck formation was affected by RDV. We observed that RDV supplement significantly reduced the LPS + ATP-induced RFP-ASC specks (**Figure 1H**). Altogether, these data indicate that RDV selectively suppressed the NLRP3 inflammasome activation in LPS-activated macrophages.

### Roles of RDV in LPS-Induced NF-κB and MAPK Signal Activation

**Figure 2:**
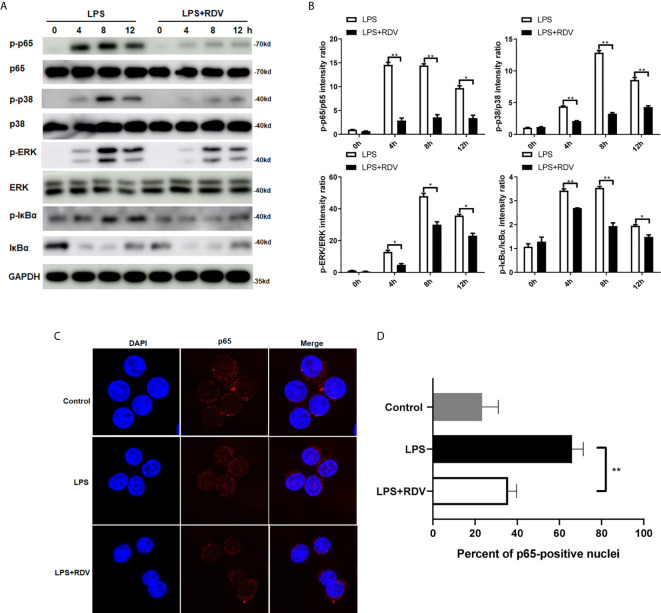
RDV suppresses the activation of NF-κB and MAPK signaling pathway. **(A)** Western blot analysis of p-p65, p65, p-p38, p38, p-ERK, ERK in mouse peritoneal macrophages under of LPS, RDV and LPS+RDV stimulation at the indicated time point. **(B)** Relative intensity analysis of p-p65/p65, p-p38/p38, p-ERK/ERK, p-IκBα/IκBα. **(C)** Macrophages were stimulated with LPS, LPS + RDV and nuclear translocation of p65 (Red) was determined. **(D)** Quantitative analysis of p65-positive nuclei. Data are presented as mean values ± SD from three independent experiments. *P < 0.05**; P < 0.01.

Following the findings, that NLRP3 level was downregulated by RDV, we next investigated whether the inflammatory signaling pathways were affected by RDV. We explored the phosphorylation levels of p65, ERK1/2, p38, IκBα in murine peritoneal macrophages. As shown in **Figures 2A, B**, LPS enhanced the phosphorylation of p65, p38, ERK1/2, IκBα in a time-dependent manner, suggesting the activation of NF-κB and MAPK cascades. However, with the supplementation of RDV, their phosphorylated levels all declined significantly. Of note, limited p65 translocation was confirmed by confocal immunofluorescence staining in the LPS + RDV group (**Figures 2C, D**), supporting the inhibited p65 phosphorylation, which was insufficient to initiate pro-inflammatory gene transcription. Collectively, these data indicated that RDV decreased the activation of NF-κB and MAPK signal cascade in inflammatory macrophages.

### Effects of RDV on LPS-Induced Pro-Inflammatory Cytokine Production

**Figure 3:**
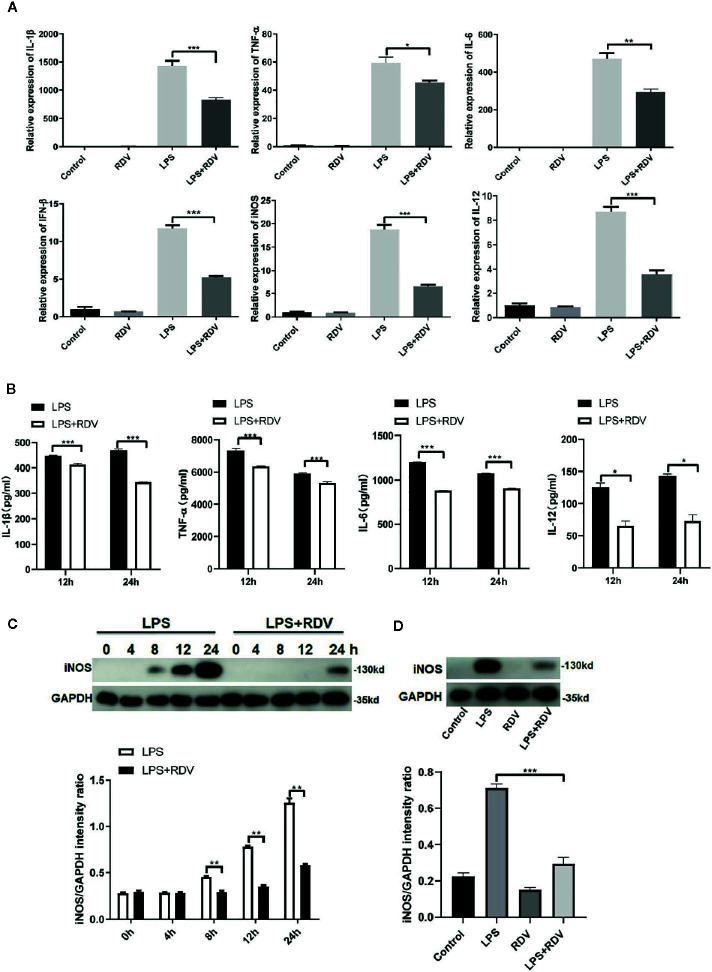
RDV inhibits the expression of pro-inflammatory cytokines. **(A)** RT-PCR analysis of TNF-α, IL-6, IL-1β, IL-12, iNOS, and IFN-β mRNA expressions in mouse peritoneal macrophages stimulated with LPS, RDV, LPS + RDV for 12 h. **(B)** ELISA analysis of TNF-α, IL-12, IL-6, and IL-1β in cell culture supernatant stimulated with LPS and LPS+RDV for 12 h and 24 h and treatment with ATP in the last 40 min. **(C)** Western blot analysis of iNOS in mouse peritoneal macrophages under LPS and LPS+RDV stimulation at different time points and relative intensity analysis of iNOS. **(D)** Western blot analysis of iNOS in the kidney and relative intensity analysis of iNOS. Data are representative as mean values ± SD from three independent experiments. *P < 0.05; **P < 0.01; ***P < 0.001.

The above data show that RDV can significantly suppress the NLRP3 Inflammasome activation and inflammatory signaling cascades activation, thus limiting inflammation. Therefore, the expression of pro-inflammatory cytokines was tested. Compared with LPS alone, the mRNA levels of TNF-α, IL-1β, IL-6 IFN-β, iNOS, and IL-12 (**Figure 3A**) and the secretion levels of IL-1β, TNF-α IL-6, and IL-12 in the cell culture supernatant (**Figure 3B**) were decreased by LPS + RDV stimulation. RDV also suppressed the expression of iNOS (M1 polarization related gene) in macrophage and in kidney tissue, indicating that RDV was able to block LPS-induced pro-inflammatory M1 polarization (**Figures 3C, D**). Thus, these data indicate that RDV suppresses inflammatory responses in macrophages by limiting pro-inflammatory cytokine expression.

### Roles of RDV in Kidney Function in LPS-Induced AKI Mice

**Figure 4:**
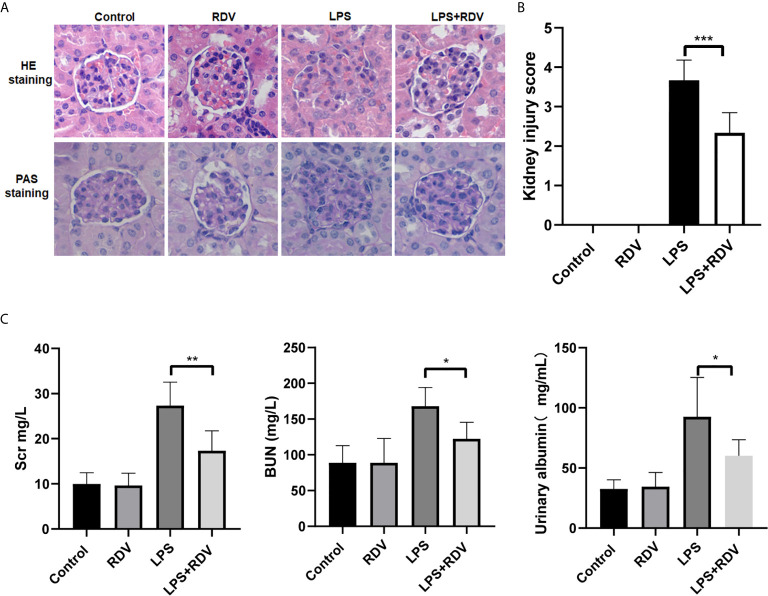
RDV alleviates LPS-induced AKI. **(A)** HE and PAS staining of kidney tissue sections. **(B)** Right panel showing the renal inflammation score based on HE staining. **(C)** SCr, BUN and urinary albumin levels detected by ELISA. Data are representative as mean values ± SD from three independent experiments. *P < 0.05; **P < 0.01; ***P < 0.001.

To determine whether RDV can rescue the kidney function in AKI, we tested the kidney injury score by HE staining and periodic acid–Schiff (PAS) staining and observed that the inflammatory foci were significantly increased by LPS challenge, consistent with the results of previous reports. However, the area of inflammatory foci was significantly reduced by the RDV supplement (**Figure 4A**) and kidney injury score was also decreased by RDV (**Figure 4B**), indicating that RDV can effectively attenuate the inflammatory and pathological state of the kidney. In addition, the kidney function of mice can be indicated by the level of BUN, SCr and urinary albumin, which can be filtered by healthy kidneys. We observed that the SCr, BUN, and albumin levels increased in LPS-induced AKI mice, but were considerably decreased by RDV treatment (**Figure 4C**). Thus, treatment with RDV can effectively inhibit the inflammatory pathological states and improve kidney function in sepsis-induced AKI mice.

### Effects of RDV on LPS-Induced NLRP3 Inflammasome Activation and Proinflammatory Cytokine Release in Kidney Tissues from AKI Mice

**Figure 5:**
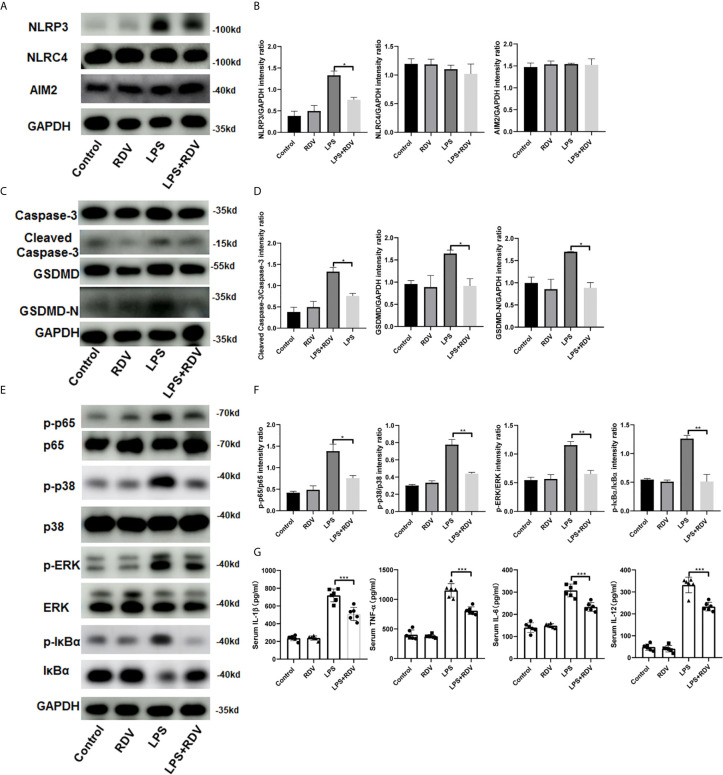
Effects of RDV on inflammatory injury in LPS-induced AKI mice. **(A)** Western blot analysis of NLRP3, NLRC4 and AIM2 in kidneys. **(B, D, F)** Relative intensity analysis of NLRP3, NLRC4, AIM2, p-ERK/ERK, p-IκBα/IκBα, cleaved-caspse-3/caspase-3, GSDMD/GAPDH, GSDMD-N/GAPDH. **(C)** Key points of apoptosis and pyroptosis pathways in kidneys were detected by Western blot. **(E)** Western analysis of p-p65/p65, p-p38/p38, p-IκBα/IκBα. **(G)** ELISA analysis of serum level of IL-1β, IL-6, TNF-α, IL-12. Data are representative as mean values ± SD from three independent experiments. *P < 0.05; **P < 0.01; ***P < 0.001.

**Figure 6:**
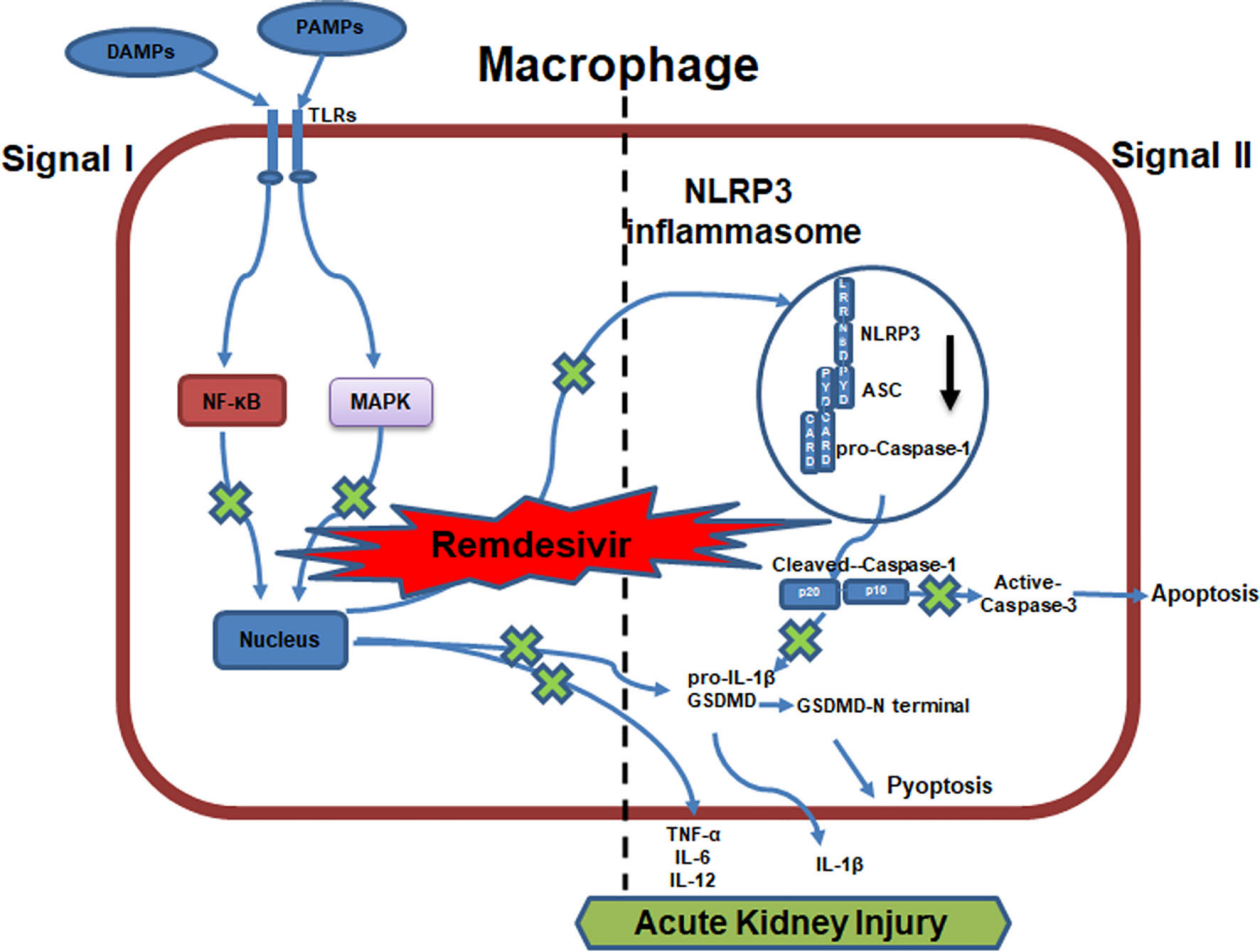
Work model for RDV alleviates AKI by inhibiting NLRP3 inflammasome activation. RDV inhibits NF-kB and MAPK signaling activation, which reduces the transcription of NLRP3, IL-1β, TNF-α, IL-6, IL-12, GSDMD inhibiting the NLRP3 inflammasome formation, apoptosis, pyroptosis and pro-inflammatory cytokine release and contributing to the alleviation of AKI.

AKI pathogenesis is accompanied by the robust production of inflammatory cytokines. By employing the AKI murine model induced by LPS, we examined the NLRP3 level and inflammatory signaling activation in the kidneys after RDV administration. RDV significantly suppressed the expression of NLRP3, whereas it had no effect on the expression of other two inflammasome adaptors, namely AIM2 and NLRC4 in the kidneys (**Figures 5A, B** **)**, supporting *in vitro* results (**Figures 1B–E**). We then explored the expression of caspase-3, cleaved-caspase-3, GSDMD, GSDMD-N in the kidneys targeted by LPS+RDV. Compared with the LPS group, the expression of cleaved-caspase-3, GSDMD, and GSDMD-N decreased significantly in the LPS + RDV group (**Figures 5C, D**). To confirm the inhibitory role of RDV in inflammatory signal way, we also detect the phosphorylation of p65, ERK, p38, IκBα in the kidney. RDV inhibits the expression of p65, ERK, p38, IκBα in the kidney (**Figures 5E, F**). Furthermore, the levels of serum inflammatory cytokines including IL-1β, TNF-α, IL-12, and IL-6 were markedly decreased by RDV compared with those in the LPS group (**Figure 5G**). Collectively, RDV may relieve LPS-induced AKI by inhibiting NF-κB and MAPK signaling, which will decrease the expression of NLRP3 and inflammatory cytokine release (**Figure 6**).

## Discussion

Given that considerable efforts have been exerted to explore the effective strategies to limit AKI but often with devastating consequences, in the present study, we investigated the effect of RDV (GS-5734), a nucleotide analog that inhibits viral RNA-dependent RNA polymerase which was authorized by the U.S. FDA as a promising therapeutic candidate for COVID-19, on AKI with LPS-induced AKI mouse model and cell culture system. NLRP3 inflammasome has a pivotal role in the pathophysiology of either obesity or diabetes by regulating metabolic inflammation (23, 24). NLRP3 inflammasome contribute to the increase in complications in COVID-19 patients with diabesity (25). Due to impaired metabolism, chronic over-activation of NLRP3 inflammasomes may lead to changes in innate immunity. The response to viral infections has become more permissive and severe (26). RDV could inhibit the NLRP3 inflammasome activation specially in our research and may play a role in the treatment of COVID-19. The two types of diseases are quite different, but similarly, they exhibit tissue-damage by the infiltration of a large number of inflammatory cells and the expression of large number of inflammatory cytokines. Although the role of RDV in the treatment for COVID-19 is controversial, we reported that it could provide protective roles in the AKI model by reducing the levels of BUN, SCr, and urinary albumin and the release of inflammatory factors, in particular the NLRP3 inflammasome, alleviating inflammation and pathological scores in kidney tissue. In addition, selective inhibition of NLRP3 by RDV mediated by the activities of NF-κB p65 and MAPK signaling, resulting in the inhibitory transcription of NLRP3 and the reduction of pro-inflammatory factors in the kidney. However, due to the short time of constructing LPS-induced acute kidney injury model, RDV has no role on LPS induced acute kidney injury by the way of subcutaneous injection of RDV post LPS intervention one hour (**Supplementary Figure 3**). We observed that the physical condition of the mice in the LPS+RDV group was better than that in the LPS group slightly before the mice sacrificing. We believe that it is better to treat mice with RDV after LPS challenge, which can effectively mimic the clinical disease process. However, the acute kidney injury model can be successfully established post LPS injection 12 h. It is difficult for us to observe the anti-inflammatory effect of the drug in such a short period of time. According to previous reports (20, 27, 28), it is acceptable to evaluate the anti-inflammatory effects for drug treatment *in vivo* with pre-treatment manner. Based on these results, RDV has the potential to serve as treatment to attenuate AKI in clinic.

Macrophages are important components orchestrating inflammatory responses that provide host immune defense (3, 5). However, excessive and continuous production of inflammatory mediators by macrophages is considered major inducers in multiple human diseases. AKI is accompanied by severe inflammatory reactions that can lead to kidney damage. The inflammation mediated by macrophages is thought to actively participate in the development of AKI (3, 29). Depletion and supplementation of macrophages can affect the inflammatory responses and outcomes of kidney injury (30). The infiltrated macrophages can not only produce a variety of pro-inflammatory cytokines/chemokines but also drive adaptive immune responses by recruiting and activating T lymphocytes, which worsen kidney injury and promote fibrosis (4). Macrophages recruited into damaged kidney tissue are polarized to pro-inflammatory (M1) type, which initiates the process of inflammatory damage or anti-inflammatory (M2) type which facilitates tissue repair in response to local microenvironment (31). According to our results, we speculated that the RDV may inhibit macrophage M1 polarization due to the reduction of M1 associated markers TNFα, IL-6, IL-1β, IL-12, iNOS in response to LPS stimulation (**Figure 3**) (**Supplementary Figure 2**), which is involved in the protective roles of RDV. However, the roles of RDV in M2 polarization including IL-10 and TGF-β production in macrophages, which exerts anti-inflammatory and tissue repair effects, need to be further addressed.

The formation and activation of inflammasome can lead to the release of caspase-1-dependent pro-inflammatory cytokines IL-1β and IL-18, and gasdermin D-mediated pyroptosis, which is a hallmark of robust inflammation in macrophages (32, 33). The dysfunction of inflammasome affects a wide range of inflammatory diseases, highlighting the therapeutic potential targeting inflammasome (34, 35). In the current study, we observed that RDV could lower the NLRP3 level, thus inhibiting the NLRP3 inflammasome formation and activation, which facilitated the protective roles of RDV against inflammation-induced kidney damage. In addition to the inhibitory effect on NLRP3, we also observed that pyroptosis and apoptosis were inhibited significantly by RDV, which indicated that RDV was able to protect macrophage against apoptosis-induced by robust inflammation. Notably, NLRP3 is also expressed in other cell types including T cells, DCs, renal tubular endothelial cells (36), in which whether RDV can inhibit NLRP3 need to be addressed in further study. Importantly, other inflammasome molecules including AIM2 and NLRC4 were not changed (**Figure 1B**) (37), suggesting the selective inhibitory roles of RDV in NLRP3 inflammasome, in which the underlying mechanisms remain unknown.

NF-κB and MAPK pathways play important roles in mediating inflammatory response. As response to upstream activation signals, NF-κB and MAPK signaling cascades are initiated to induce downstream inflammatory activities, which trigger the transcription of targeting inflammatory genes. The NF-κB pathway comprises p65/p50/c-Rel subunit-involved canonical signal and p100/p52/RelB-involved noncanonical signal. NF-κB is involved in the regulation of NLRP3 transcription in TLR-stimulated macrophages through binding to the NF-κB sequences in the NLRP3 promoter (38). Bruton’s tyrosine kinase (BTK) regulates NF-κB and the NLRP3 inflammasome specifically in macrophages, the *in vivo* cellular target of ibrutinib (24). Here, we observed that RDV down-regulated the activities of NF-κB p65 in particular its nuclear translocation in macrophages upon LPS stimulation (**Figure 2C**), which is consistent with a previous report which showed that NF-κB p65 is involved in pro-inflammatory macrophage activation (39). Intriguingly, RDV is able to repress NF-κB activation during a long term (up to 12 h) indicating its protect roles in the chronic inflammation. However, NF-κB overexpression or stabilization assay is required in further studies to confirm the roles of NF-κB activity by RDV in NLRP3 transcription. In addition to NF-κB signaling, NLRP3 promoter activity may be modulated by another factors. Thus, further efforts, involved in RDV-regulated NLRP3 expression, may be exerted to investigate.

In conclusion, we provided evidence that the treatment with RDV can protect mice against LPS-induced AKI by specially inhibiting NLRP3 inflammasome activation in macrophages, thereby reducing inflammation-induced renal damage and improving the recovery of renal function. Importantly, the inhibited NLRP3 inflammasome by RDV was mediated by impeding NF-κB and MAPK signal thus suppressing the expression of inflammatory genes including NLRP3. RDV administration significantly decreased the inflammatory response and improved the kidney pathological score, which resulted from the suppression of NLRP3 inflammasome activation by impeding NF-κB signal activation. Our study thus uncovered the feature of RDV in AKI treatment and mechanisms underlying RDV-regulated NLRP3 inflammasome activation and innate immune responses. The results suggest that RDV has potential as a therapeutic option for controlling the development of AKI.

## Data Availability Statement

The original contributions presented in the study are included in the article/**Supplementary Material**. Further inquiries can be directed to the corresponding authors.

## Ethics Statement

The animal study was reviewed and approved by the Laboratory Animal Ethical Commission of Shandong Provincial Hospital Affiliated to Shandong First Medical University.

## Author Contributions

LY, FK, and SZ designed the study. LY, YD, and HJ performed the experiments. LY, YL, and HYZ interpreted the data. LY, HXZ, and FK wrote the manuscript. All authors contributed to the article and approved the submitted version.

## Funding

This work was supported by the National Natural Science Foundation of China (81970578), Academic Promotion Programme by Shandong First Medical University (2020LI001).

## Conflict of Interest

The authors declare that the research was conducted in the absence of any commercial or financial relationships that could be construed as a potential conflict of interest.
