# Supplementary material for: Remdesivir Alleviates Acute Kidney Injury by Inhibiting the Activation of NLRP3 Inflammasome
Source: Front Immunol. 2021 May 21;12:652446. doi: 10.3389/fimmu.2021.652446 (PMC8176923; doi:10.3389/fimmu.2021.652446)
Supplement: Supplementary file 1 [file DataSheet_1.docx]

**Supplementary Data**


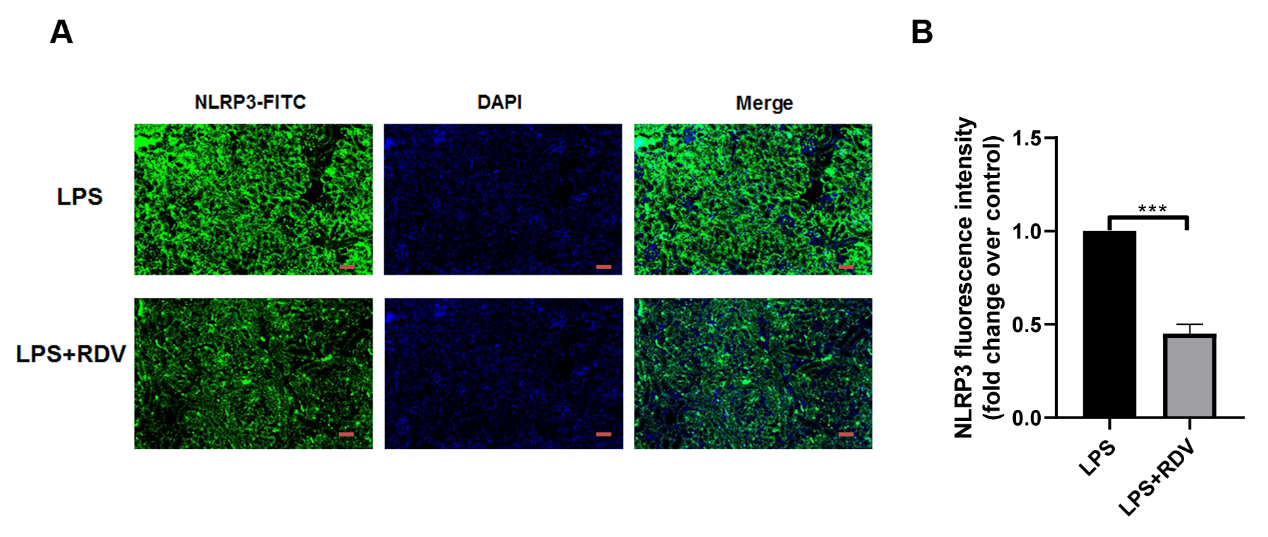
**Figure 1. Effects of RDV on NLRP3 expression in LPS induced AKI animal model.** A. The expression of NLRP3 was detected by immunofluorescence (Magnification, 200X; scale bar, 100 μm). B. Fluorescence intensity analysis. ***P < 0.001.


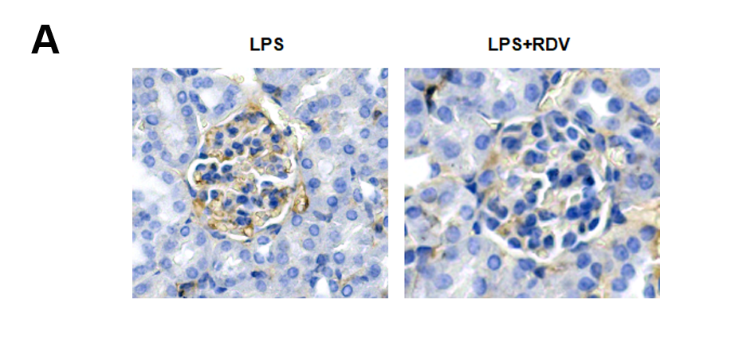


**Figure 2. Effects of RDV on macrophage infiltration in LPS induced AKI animal model.** A. Roles of RDV on macrophage infiltration in LPS induced AKI animal model. Immunohistochemistry localized F4/80 expression to macrophages.


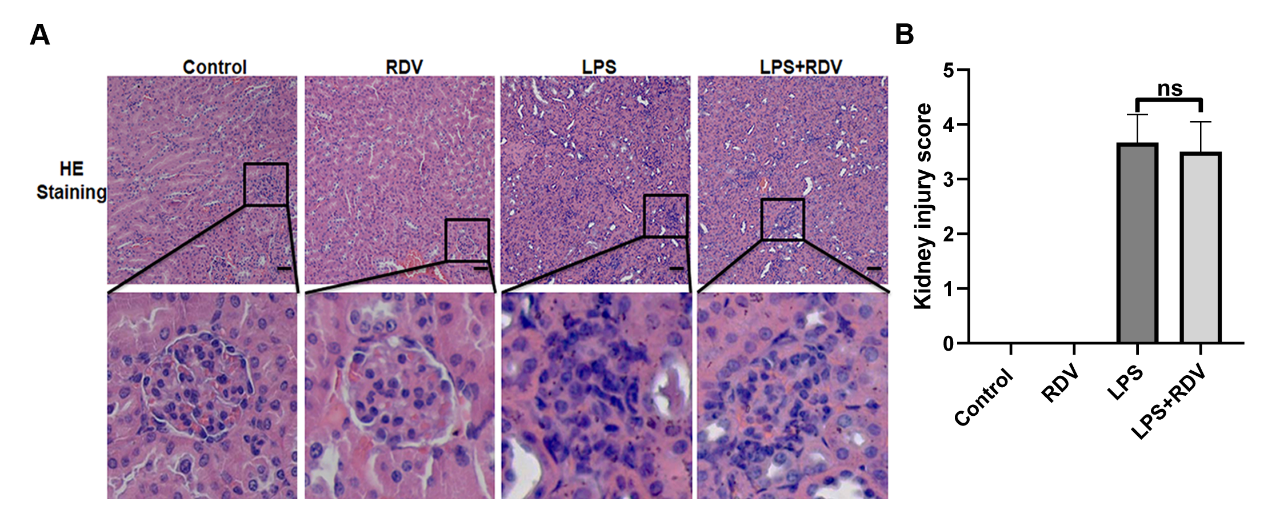


**Figure 3. RDV has no role on LPS induced acute kidney injury by the way of subcutaneous injection of RDV one hour post LPS challenge.** A. HE staining of kidney tissue sections(Magnification, 200X; scale bar, 100 μm). B. Right panel showing the renal inflammation score based on HE staining. ns: no significant differences.
